# Parents' Experiences of Receiving Professional Support Through Extended Home Visits During Pregnancy and Early Childhood—A Phenomenographic Study

**DOI:** 10.3389/fpubh.2021.578917

**Published:** 2021-02-22

**Authors:** Caroline Bäckström, Stina Thorstensson, Jessica Pihlblad, Anna-Carin Forsman, Margaretha Larsson

**Affiliations:** ^1^School of Health Sciences, University of Skövde, Skövde, Sweden; ^2^Regionhälsan Midwifery Unit, Västra Götalandsregionen, Sweden

**Keywords:** mother, father, labor, parental transition, child health care nurse, midwife, nurse, social service

## Abstract

**Background:** While becoming a parent can be challenging for all, it can particularly be challenging for those parents and children who are in a vulnerable situation—e.g., in families whose members have problems related to health, relationships, or socioeconomic status. It is essential for health care professionals to identify the more vulnerable families at an early stage. Home visits are one cost-effective way of identifying and supporting such families. This study describes the parental experiences of an intervention that involves professional support in the form of extended home visits. The aim of the study is to describe the parents' understanding of their experiences of receiving professional support through extended home visits both during pregnancy and the first 15 months of their child's life.

**Methods/Design:** A phenomenographic approach was used. Semi-structured interviews were conducted with 12 parents who had received the intervention. The interviews were analyzed using the seven-step phenomenography model described by Sjöström and Dahlgren.

**Results:** The following three descriptive categories emerged from the analysis: (1) *conceptions concerning the meaning of the physical environment*, (2) *conceptions concerning extended home visits promoting feelings of self-confidence in the parental role*, and (3) *conceptions concerning extended home visits promoting parental participation and relations*.

**Conclusion and Clinical Implications:** Extended home visits as a form of professional support appear to promote parental self-confidence in parenting ability, giving parents a feeling of security that facilitates conversation with professionals. Children and their entire families had natural roles during home visits, which allowed the children to behave more characteristically. Furthermore, the home visits were understood to facilitate social support through social activities at the child health center as well as integration into Swedish society for migrant parents. Professional support should be adjusted to the unique individual needs of parents, which demands a variety of supportive interventions—for example, reorganizing one or two of the regular clinical visits currently being scheduled as home visits instead.

## Introduction

While being a parent can be challenging for anyone, it is especially so for parents and children who are in a vulnerable situation—for example, in families whose members have problems related to health, relationships, or socioeconomic status. In Sweden, there is free professional support available for expectant and new parents at both individual and group levels. Professional support is provided for parents and their children during pregnancy and until the child's school age. Access to high-quality family planning services is fundamental for realizing the rights and well-being of women, men, and children (1). The antenatal and child health service program in Sweden is based on the Conventions on the Rights of the Child (2), maintaining a focus on the child perspective and the child's best interest.

It is essential to identify the more vulnerable families at an early stage. Home visits represent a cost-effective way of identifying and supporting such families (3–5). Home visits can help strengthen relationships between parents and children, children and child healthcare (CHC) nurses, and parents and CHC nurses (6). Home visits provide opportunities for professionals to pay attention to the environment in which a family lives in, making it possible for them to increase their understanding of the family and to support the family's unique needs (7). Furthermore, extended home visits can strengthen parental knowledge, skills, and motivation related to parenting and are favorable for the family's health and function—supporting children during both childhood and adolescence (3). Previous research has reported that home visits provide early, individual, and family-centered support (8), which strengthens both the self-confidence and social networks of parents (9). For instance, it has been found that parental confidence, knowledge of social services, and local family resources can be strengthened for migrant fathers (10). In addition, home visits have been shown to reduce the incidence of child abuse (11).

Most expectant parents have both positive and negative feelings connected to the role changes encountered during the transition from one stage of life to the next (12). Seay et al. (13) defined positive parenthood as a continuous relationship between parents and children that includes care, nurture, teaching, training, and leadership as well as communication that consistently and unconditionally secures the needs of the child. The transition to motherhood is considered to be a pivotal and paradoxical life experience for women, which entails the processing of existential questions such as what the meaning of life is and what makes life worth living (14). Men's transition to fatherhood seems guided by the social context in which they live and work as well as by their personal characteristics. Men struggle to reconcile their personal and work-related needs with those of their new families (15). Expectant and new fathers may lack relevant information, role models, or guidelines to help them during the transition to parenthood and the fathering role (16). Resilience refers to a person's long-term ability to handle change and continue to develop, which is of great importance in parenting (17). Bäckström et al. (18) reported that childbirth and the transition to parenthood have a positive impact on the abilities of parents to cope with life.

In Sweden, midwives are independently responsible for managing healthy pregnancies (19). Midwives at antenatal units offer expectant parents six to nine antenatal visits (20), which can be described as health check-ups for detecting any pregnancy-related complications (19). Moreover, individual support needs of parents are meant to be identified during these visits, especially in terms of parents who are showing signs of mental and social illness, which may make it challenging for them to fulfill their upcoming parental role (19).

When a baby is born, its family is entitled to CHC. In Sweden, this area is staffed by professionals, such as child health nurses, who provide families with access to physicians, psychologists, dieticians, social workers, and speech therapists (21). The goal is to promote child health by providing health examinations, guidance, and vaccinations for all children as well as parental support. Additional goals are to monitor the mental health of parents, strengthen social relationships in the family, and facilitate responsive parenting (22). Child health nurses first meet the children and their parents upon birth and the families receive a total of about 13 visits throughout the first 6 years of their children's lives. Of these visits, two are home visits (the first one and the one that occurs when the child is 8 months old), while the remaining visits take place at a CHC center.

In most Western countries, professional parental support is offered to expectant parents within antenatal care and to new parents within CHC. Professional support—promoting the parental role construction—provides guidance for the parents and a safe growing environment for the children while also developing a trusting relationship and can facilitate the transition to parenthood (23). Bäckström et al. (24) reported that professional support contributes to expectant parents' mental preparedness for both childbirth and parenting. In a review of effective new-parenting interventions, Gilmer et al. (25) showed that multiple approaches are of value when they allow expectant and new parents' access to information or education at a time and in a format that suits them. In Sweden, there are also so-called family centers that offer professional support coordinated at the central level in the municipality. It is a collaboration that includes open preschool, antenatal health, CHC, and social services focused on promotion (22). Open preschool is a unique form of preschool because the children attend it with their parents, it costs nothing, and no prior registration is required. Visitors choose when they want to come and go. The organization of the family centers is based on a national strategy for community assistance and is intended to support parents in their parenthood (26). The Swedish social service is a municipal administration ordered by the municipality's social committee and regulated by the Social Services Act [(27): 453]. The primary purpose of social services is to promote the foundation of democracy and solidarity, as well as people's economic and social security, equality in living conditions, and active participation in social life [(27): 453]. Family supporters working in social services are responsible for protecting children from growing up in poor conditions and must do so through outreach activities.

In Sweden, the goal is to create societal conditions for good and equal health throughout the population [(26): 131]. However, there are substantial health inequalities in the country (28). Children's health and well-being are affected, for example, by their home environment, parents' health, and social relationships (29). Today, antenatal and CHC services reach almost all pregnant women and parents with a newborn child in Sweden—nevertheless, some parents need more professional support. Welsh et al. (29) showed that interventions in family settings are successful in building the strengths of children and supporting parenting, which may have positive outcomes on child health, both universally and within disadvantaged groups. During the first 15 months of a child's life, the parental transition to parenthood is shaped (30). From a health equity perspective on early childhood development, Barboza et al. (4) reported that home visits can strengthen the roles and relations within a new family unit. Pålsson et al. (31) argued that cross-sectional collaboration between professionals possessing complementary knowledge may be one way to resolve issues lacking topical skills. Furthermore, introduction to different professionals could increase the parents' awareness of how professional support can be accessed both before and after their baby is born. To the best of our knowledge, there is a lack of research on how the skills and experiences of expectant and new parents can be strengthened during their parenthood *via* collaboration with professionals in antenatal health, CHC, and social services. Hence, this study aimed to describe the parents' understanding of their experiences of receiving professional support through extended home visits during pregnancy and the first 15 months of their child's life.

## Methods and Design

This study was derived from an intervention initiated in the *Reinforced Parenting—Extended Home Visits* project. This intervention included professional home visits to parents during pregnancy and the first 15 months of parenthood. It is described in more detail below.

In order to answer the aim of the study, we used a qualitative method and an inductive and phenomenographic approach. Qualitative research is used to gain an understanding of how groups of people describe a phenomenon. Data are usually collected in a natural setting that is sensitive to the people and places under study (32). Both deductive and inductive approaches can be used in qualitative research. For the current study, an inductive approach was employed to allow the facts to be acquired from the participants' narratives (33). Phenomenography is suitable when conducting research on a broad range of various experiences for a specific phenomenon (34). Using the phenomenographic approach allows for investigating the different ways in which people make sense of, experience, and understand phenomena. The intention is to discover the underlying structure of variance in the perceptions of a phenomenon rather than the phenomenon's core—the latter being the focus of other qualitative research methods (34). The phenomenon examined in this study was parental understanding of their experiences of receiving professional support through extended home visits during pregnancy and the first 15 months of parenthood.

### Intervention

For the intervention, public antenatal, CHC, and social services introduced extended home visits for parents in two regions in southwestern Sweden. This home visit service was conducted by professionals—e.g., midwives working within antenatal care, district nurses working in CHC, and family supporters working in social services. The two regions were chosen for the intervention because they generally included parents from a lower socioeconomic status and with higher professional support needs that were determined according to the Care Need Index (35), which is a social deprivation index. In the two regions, there were also well-functioning family centers, which provided a valuable aspect for the intervention. These family centers included antenatal and CHC centers and social services that practiced mutual cooperation. The development of the intervention was based on the Swedish national strategy for community support and assistance to parents in their parenting [(26): 131]. The aim of the intervention was to strengthen the self-confidence of parents and to promote their trust in antenatal care and CHC as well as social services. Furthermore, the intervention aimed to promote equality and participation among the parents and to identify families with extra professional support needs at an early stage.

The intervention consisted of extended home visits for expectant and new parents. Participants were consecutively selected for the intervention because the recruitment process was tailored to a specific period (May 2018–May 2019). In total, 100 children were estimated to be born within the setting during the specified time period and, therefore, 100 families were targeted for the intervention. The inclusion criteria were that the parents lived in one of the two included regions and that they were expecting their first child to be born in Sweden (i.e., the parents could have had another child previously in another country). Midwives who worked at antenatal wards within the regions asked the parents who met the inclusion criteria about their willingness to participate in the intervention during a prenatal assessment between the 29th and 32nd week of pregnancy. However, the targeted number of families was not recruited because fewer than 100 children were born in the setting during the chosen period. In addition, the midwives provided different reasons because of which they did not ask certain families about their willingness to participate—e.g., heavy workload or forgetting to inform families about the study. In total, 70 families were informed about the intervention and were invited to participate; 20 refused and 50 agreed to participate. Some of the families that declined to participate explained that they were not in need of professional support in the form of extended home visits. The intervention took place from 2018 to 2020 and the extended home visits provided to the parents participating in the intervention are described in detail in Table 1.

**Table 1:** Overview of the intervention consisting of extended home visits for parents during pregnancy and the first 15 months of parenthood.

**Extended home visit**	**Time point**	**Professionals responsible for the extended home visit**	**Issues brought up during extended home visit**
1. To become a parent	Pregnancy week 34	Midwife working within antenatal care, and family supporter working within social services	Relation to and support from partner; social network such as family, friends and significant others; the expected child and her/his needs; first time at home after birth; parenthood; emotional changes/reactions; breastfeeding; and skin-to-skin contact.
2. To meet and receive the child	Two weeks after birth	Child health nurse working within child health care, and family supporter working within social services	The child's sleeping position; safety of the child; the child's eating; how to comfort a sad child; the parent's health and well-being, alcohol and tobacco use; the parental couple relationship; violence in close relations; the parent's social network; child health service and professional caregivers available for the family
3. To be together	Four months after birth	Child health nurse working within child health care, and family supporter working within social services	The child's well-being; routines for the child's eating and sleeping, and language development as babbling and laughing; safety of the child; infections and self-care; the library and children's book; the parent's health and well-being, alcohol and tobacco use; the parental couple relationship; violence in close relations; social medias; child health care and professional caregivers available for the family
4. To lead and follow	Eight months after birth	Child health nurse working within child health care, and family supporter working within social services	The child's language development; daily routines and feelings of security; safety of the child; infections and self-care; dental care; kindergarten/preschool; parental leave; the parent's health and well-being; violence in close relations
5. To be a family	Fifteen months after birth	Child health nurse working within child health care, and family supporter working within social services	The child's eating and sleeping-routines; the child's communication, language development, and digital screen time; what the child likes to do; infections and self-care; the parental couple relationship; the parent's time for own interests; violence in close relations

The intervention included five home visits between the 34th week of pregnancy and 15 months after birth (Table 1). A family supporter working for a social service center participated in each home visit. The five home visits included in the intervention differed from standard care in the following manner:

1. The first home visit (in 34th week of pregnancy) was conducted by a midwife in addition to standard care, while the subsequent four home visits were conducted by a child health nurse;
2. In standard care, parents are provided a home visit 2 weeks after birth—for the intervention, a family supporter working for social services was present during the standard care home visit 2 weeks after birth;
3. At 4 months after birth, the parents who were included in the intervention were provided a home visit instead of visiting a CHC unit, which is included in standard care;
4. In standard care, parents are provided a home visit 8 months after birth—for the intervention, a family supporter working for social services was present during the home visit 8 months after birth;
5. At 15 months after birth, the parents who were included in the intervention were provided a home visit instead of visiting a CHC unit, which is included in standard care.

Parents who declined to participate in the intervention were offered standard care.

### Settings and Participants

The current study was carried out in two regions in southwestern Sweden, which have a population of ~15,000 inhabitants. Parents who met the inclusion criteria and parents who had participated in the *Reinforced Parenting—Extended Home Visits* project were informed about the current study and invited to participate. In total, 40 parents had completed the intervention at the time of recruitment and 12 (transgender) parents agreed to participate in the current study (eight females and four males). The participating parents were 23–57 years of age, seven had a university education, and four were born in Sweden, while the rest were born in Syria, Cameroon, or Morocco. The number of children that the participants had ranged from one to seven. Nine of them were first-time parents, while the others had two to seven children each. The participants comprised parents who participated individually from a parental couple and parental couples in which both parents participated. The participants represented a broad variety of parents who had knowledge about the topics of interest for this study. Therefore, the number of participants was deemed to be satisfactory.

### Data Collection

The responsible managers at public CHC centers gave written approval for parents who took part in the intervention to be asked about their interest in participating in interviews for the current study. A semi-structured interview guide was created, which was initially tested in two pilot interviews in order to explore informant interpretation of the questions. After the pilot interviews, one question was revised to increase clarity. The two pilot interviews were included in the data analysis because the interviewees met the inclusion criteria. Examples of the questions included within the interview guide are: *Could you describe your experiences with the extended home visits? What meaning have the extended home visits had for you?* Examples of follow-up questions are: *Could you describe this further? Could you give an example?* All the interviews were conducted between January and March 2020; three interviews were held with the parental couple, while the other interviews were held with individual parents. The participants could choose whether they wanted to be interviewed individually or with their partner. They were also able to choose the interview setting—in their homes or at the CHC center. Two of the authors (ACF and JP) carried out the interviews. Seven interviews were conducted in Arabic with a professional translator, one was conducted in English, and the rest were conducted in Swedish. The interviews lasted 22–64 min and were all recorded digitally and transcribed verbatim (the transcribed interviews total 66 pages in A4 format). From the interviews conducted in Arabic with a professional translator, the transcriber transcribed the Swedish words. The interview conducted in English was transcribed in English.

### Data Analysis

Data analysis was performed by three of the authors in accordance with the phenomenographic approach (34) and the seven-step model described by Sjöström and Dahlgren (36). In the first step (*familiarization*), the transcribed interviews were read several times. Subsequently, the narratives from all participants concerning their experiences of receiving professional support through home visitations during pregnancy and the first 15 months of parenthood were gathered into statements, representing the second step (*compilation*). In the third step (*condensation*), the statements were concentrated to obtain a representative description of the participants' experiences. Similar statements were grouped together in the fourth step (*grouping*), while the groups were compared to find similarities and differences in the experiences in the fifth step (*comparison*). In the sixth step (*naming*), the conceptions and emerging descriptive categories were named, while the logical relationship between the descriptive categories was analyzed in the seventh step (*contrastive comparison*), resulting in a hierarchical arrangement among the categories that was presented as an outcome space. Three of the authors (CB, ACF, and JP) initially participated in each step of the analysis process. Thereafter, all authors discussed the analysis and the different steps of the analysis process were repeated to verify the results. All authors contributed with their experiences of qualitative research when discussing the analysis to reach a consensus. The analysis and interpretation of the results were also discussed continually among a group of MSc students during the analysis process. All authors participated in the process of writing the text. The parents' socioeconomic status, gender, birth country, and parity were not generally considered when conducting the analysis—however, when the results showed any participant understanding that only represented a specific group of participants (e.g., mothers/fathers, first-/second-time parents), this was clarified in writing in the results section. Overall, the authors agreed that the participants represented a diverse group of individuals regarding the characteristics mentioned. All data were analyzed in Swedish, except for the interview transcribed in English which was analyzed in English. The authors CB, ACF, and JP checked the transcriptions and the recordings were available during the analysis. No computer program was used for the analysis.

### Ethical Considerations

The participants received both written and verbal information about the study before they provided their informed consent to participate. Prior to the interviews, the participants could ask questions about the study. This was done to ensure that the aim of the study was understood and that participation was voluntary. The study complied with Swedish law and was approved by the Regional Ethical Review Board in Gothenburg (Dnr 2019:03906).

## Results

The interviews with the participants were analyzed. Three descriptive categories emerged with their attendant conceptions of receiving professional support through extended home visits during pregnancy and the first 15 months of parenthood (Table 2).

**Table 2:** Overview of descriptive categories and conceptions.

**Descriptive categories**	**Conceptions**
Conceptions concerning the meaning of the physical environment	The home environment is a safe place that facilitates conversation The home environment creates conditions for the child to have a more natural place in the meeting
Conceptions concerning extended home visits promoting feelings of self-confidence in the parental role	Extended home visits facilitate professional support that reassures the parental role Extended home visits facilitate professional support that is based on the individual
Conceptions concerning extended home visits promoting parental participation and relations	Extended home visits facilitate a close and equal relationship with the professionals Extended home visits facilitate parental participation, togetherness, and integration

### Conceptions Concerning the Meaning of the Physical Environment

The participants conceptualized that, for the extended home visits, the physical environment—the participants' home—was meaningful. This was because they considered their home to be a safe place, which affected the entire family and facilitated conversation with professionals. Furthermore, the home environment affected both the content of the professional support and the participants' conceptions of it. The participants described that the professional support received during the extended home visits facilitated feelings of manageability and meaningfulness as well as a sense of coherence. The first descriptive category consisted of the following two conceptions: (1) *the home environment is a safe place that facilitates conversation* and (2) *the home environment creates conditions for the child to have a more natural place in the meeting*.

1) The Home Environment Is a Safe Place That Facilitates Conversation

The participants conceived the home environment to be a safe place that facilitated their conversation with professionals. The home environment enabled the participants to feel calm and behave more naturally because their home was their natural place in comparison to antenatal or CHC centers or social services, which were considered to be the arenas of healthcare professionals. From the statements, it was clear that the meetings with the healthcare professionals tended to be more relaxed during home visits than at healthcare centers. In addition, the home environment facilitated a more personal feeling, which allowed for more open and permissive conversation. In contrast, meeting professionals at healthcare centers was described as more inflexible and official, which limited the participants' conversation. Altogether, the home environment was conceptualized to convey a calm that allowed the participants to behave more naturally, honestly, and spontaneously—making it easier for them to remember what issues they wanted to discuss. This conception was common among the participants. They described it as a prerequisite for them to be able to receive professional support regarding sensitive or difficult issues, as pointed out by one of the fathers (first child):

*When they come to meet me at home, it feels more open. It is more…it is the environment and it is mine, I feel at home.…It is easier to answer questions, it is easier to open up, and it is easier for my wife, who thinks it is easier to open up, sort of, when it [the care meeting] occurs at home. That is what I mean by “positive,” that it is nice in that manner*.

The home environment also allowed for discussions to take place that felt more current at the moment of the care meeting. The participants conceptualized that the conversations with the professionals were more extensive and detailed. They had opportunities to show the professionals things and to talk about what was essential for them in the family's natural environment. The participants described that extended home visits allowed them to receive professional support in line with the family's contextual circumstances, which facilitated feelings of manageability. This was the case for both first-time parents and parents with other children, corroborated by one of the first-time mothers:

*It is good.…You remember what you wanted to ask about issues when you are at home and visually see everything*.

Furthermore, one of the mothers conceived that home visits provided opportunities for professionals to investigate a child's home environment, allowing them to detect children who are in potential danger:

*I also think it's good that the family supporter is involved and that they come home and see a little how it is at home because then, maybe, it is possible to discover if something is not right. Now the visits are scheduled, so that you can prepare your home, but, still, it may be possible to notice a little more warning signals…I am thinking of children who may be in danger…There are four visits instead of two, so you can see it a little easier, maybe, hopefully*.

2) The Home Environment Creates Conditions for the Child to Have a More Natural Place in the Meeting

The participants conceptualized that the home environment created conditions for the children to have a more natural place in the meeting. During the extended home visits, the children were in their natural environment and, consequently, acted more genuinely. One of the participants described that she let her child play on the floor during the home visits, which she did not allow the child to do during visits at the CHC centers:

*It felt somehow better at home because, when you are at the child healthcare center, you do not let her down on the floor in the same way and she is not allowed to crawl around but, at home, she can lie on the floor…and then they [the professionals] can see…how she moves, compared to when she sits in the baby seat*.

Moreover, the entire family (parents, child, and potential siblings) was seen in its natural environment, making the lives of each unique family more visually evident for the professionals. This was especially so for participants with other children. The participants who had other children conceptualized that siblings had a more prominent role in the conversations with the professionals during home visits than during the meetings at healthcare centers. The needs of the entire family became more visible and every individual in the family was given the same important role as the new child, which facilitated feelings of meaningfulness. A non-Swedish-speaking participant described this as follows:

*During the first visit, we sat there, my wife and I. They [the professionals] started to tell us how we should handle the newborn child, how we should think regarding the big brother, considering his sensitivity. How we should handle him and how we should, we should see that both children are feeling ok. So, not just the baby. That was important information for us*.

The participants described the value of the extended home visits in allowing the professionals to evaluate the children's development in their natural environment. Children were described as showing their true selves to a more considerable extent during the home visits than at visits taking place at healthcare centers. The participants conceptualized that the children played more easily, naturally, and spontaneously; they were not hindered from doing so during the home visits. This facilitated feelings of security among the participants because they knew that the children had been given the opportunity to show their real development. One first-time mother described it as follows:

*So, I can imagine that it is more fun for the child, too. That [the child] feels safer. That feels good [for me]. It is really crucial for me that she feels safe, then it becomes more fun if it is possible to have meetings like this, meetings in which she is extra safe*.

### Conceptions Concerning Extended Home Visits Promoting Feelings of Self-Confidence in the Parental Role

The participants conceptualized that extended home visits allowed professional support that was reassuring and based on the individual. As a result, they felt that their self-confidence in the parental role increased. The second descriptive category contains the following two conceptions: (1) *extended home visits facilitate professional support that is reassuring* and (2) *extended home visits facilitate professional support that is based on the individual*.

1) Extended Home Visits Facilitate Professional Support That Is Reassuring

The participants conceptualized that, during the extended home visits, their parental role was put in its real context and, hence, made more visible to the professionals. The professionals reinforced the function of the home environment and the parenthood provided within it. The participants conceptualized that their daily lives were naturally linked to their parenthood. From this, the professionals could see how the participants' parental role worked for both them and their children, which was considered to be valuable. One of the non-Swedish speaking mothers described this in the following manner:

*They also showed me how to put the baby to bed in the first weeks after birth. She [the child] was very sad and just cried at night. They told me: “When you put her on your stomach or next to your chest, she hears your heartbeats”…it was very good and it helped. She slept well afterward*.

The extended home visits facilitated the participants' abilities to take in the advice and information provided by the professionals. From the statements, it was clear that—regardless of their gender, Swedish language skills, or number of children—the participants felt that they were seen and cared for as unique individuals. Furthermore, they conceptualized that home visits allowed professional support, which was reassuring and strengthened their parental role. One of the fathers described this:

*I feel happiness due to the feeling of security. Moreover, that you feel calm, that it will be ok. There is help to receive. You feel that other persons…will engage with our child. They will help us. They are there for us. We can turn to them when needed*.

2) Extended Home Visits Facilitate Professional Support That Is Based on the Parent as an Individual

The participants conceptualized that extended home visits provided room for their individual needs and that the professionals' agenda did not overshadow their needs. Furthermore, they described that extended home visits allowed for a calmness that made it possible for them to share their thinking; also, when they asked questions, there was time for the professionals to listen and answer. The home environment made it possible for the professionals to provide support based on the participants as unique individuals and on the specific requirements that their families had. During the home visits, the professionals brought up issues that were related to the real situation that the family was currently experiencing. One of the participants described, for example, how the professionals recognized the family's dogs during a home visit:

*She got to see our dogs and talk a little about them and so on. It [the conversation] was on another level, perhaps*.

Home visits also allowed the participants to illustrate points in their home practically (a changing table, for example) and the information that the professionals gave verbally was strengthened when they also showed the parents how to handle an issue (a diaper change, for example). This led to the participants perceiving that extended home visits facilitated their feeling of being well-informed so that they felt prepared, calm, and secure. This was especially so among the non-Swedish-speaking participants, as one of the mothers related:

*What was good, really important for me, was that we spoke. She spoke beforehand, gave me information beforehand, so that all information I received was ahead of the visit, so that I felt prepared…That felt important for us, it gave my family and me everything we needed without having to ask for it.so it was super complete*.

### Conceptions Concerning Extended Home Visits Promoting Parental Participation and Relations

The participants conceptualized that the extended home visits facilitated their participation within the care meeting, their relations with the professionals, and their other social connections (their partner or other parents). The third descriptive category covers the following two conceptions: (1) *extended home visits facilitate a close and equal relationship with professionals* and (2) *extended home visits facilitate parental participation, togetherness, and integration*.

1) Extended Home Visits Facilitate a Close and Equal Relationship with the Professionals

The participants conceptualized that the extended home visits enabled a closer, more personal, and more equal relationship between them and the professionals. The relation to the professionals was described as closer because home visits enabled care meetings to be conducted on a more personal level. This was described as essential by the participants who did not have their families or relatives within a close geographic area:

*It felt like personal; it was like a family member coming home to me, a cousin, a family member who asks how I feel…and provides me with support…I have no one here. I have no contacts…It is not easy…It was good for me*.

Those participants conceptualized their relationship with the professionals as complementing the absence of their family and social network in some manner. Such a close and personal relationship was seen as valuable support that facilitated feelings of security and togetherness, which was subsequently valuable for them in their parental role:

*That was strengthening for us because we are alone in Sweden; we do not have any social network. We do not have our parents here. We are new to this country, so it has been a great support…how to say it, emotionally.…So, it has been good support for us since we are alone here*.

The participants also conceptualized that the home visits enabled them to meet the professionals on a more equal level than at healthcare units. By “equal level,” the participants were referring to the social equality condition—finding themselves to have the same status and respect as the professionals. This occurred because the professionals came to visit them in their homes, arriving as guests, which established a feeling of the visit being a meeting of equals between the participants and the professionals. The participants also described care meetings at the healthcare units as being implemented with professional authorization that caused an imbalance, where the participant was at a disadvantage. The feelings of equality that arose during home visits made it easier for the participants to ask questions that were perceived to be uncomfortable—questions they may not have otherwise dared to ask at the healthcare center. The participants described that the healthcare centers functioned as a barrier during the care meetings because the participants felt like they were meeting healthcare professionals as authorities in these cases. In contrast, the participants conceptualized that, during home visits, they were meeting a person. This was a common conception among the participants, described by one of the mothers:

*It becomes a bit different, when you come into a healthcare environment at the primary healthcare center, it is a bit more…she has working clothes on then and behaves differently, and it is more apparent that she has a professional role in some manner. When she comes to the house, she has ordinary clothes on and it becomes more like if you have a meeting with a person, different, I would say. Yes, it feels more personal*.

The participants also described that they did not distinguish between the professionals regarding their assignment. Instead, the participants conceptualized the professionals—e.g., midwives working within antenatal care, district nurses working within CHC, and family supporters working within social services—as a cohesive group or as a whole.

2) Extended Home Visits Facilitate Parental Participation, Togetherness, and Integration

The participants described that the home environment provided them with opportunities to participate in the meeting with the professionals to a more considerable extent in comparison to meetings with the professionals at the healthcare center. These feelings of participation were based on the feeling of being seen as an individual. In addition, the participants conceptualized a feeling of not only receiving professional support but also of having the opportunity to ask for support more easily than during a care meeting at the healthcare unit. Thus, the participants expressed that they felt free to ask for further support from the professionals during the home visits. A common understanding among the participants was that they felt free to ask for extended professional support, such as extra appointments with family supporters working within social services, during the home visits:

They have told me: “Whenever you need to ask us something, if you need advice or support, we are always here.”

Moreover, a non-Swedish speaking mother described participating in activities for new parents, arranged by professionals at the healthcare unit, to a larger extent after receiving the home visits:

*We talked about different activities that we could participate in…they had an arranged activity in the library in which children of all ages could participate. They could listen to someone reading a book. There was also another activity where mothers gather and talk and the children play together…They told me about it, it was something new, and I learned from it*.

As a result, the participants described feeling that society engaged with them and provided them with the support they required. The participants who had a migration background conceptualized that the home visits facilitated their integration into the Swedish society because they learned about Swedish society and how to manage parenthood in Sweden.

*It is a feeling that society engages with you, that it cares for you. That they will help you. It facilitates the integration into society and you feel welcomed—that you are being supported, that you are able to become integrated. You feel togetherness somehow. You feel so comfortable, relaxing in your home, and they come home to you. So, it makes it more accessible*.

### The Outcome Space

**Figure 1:**
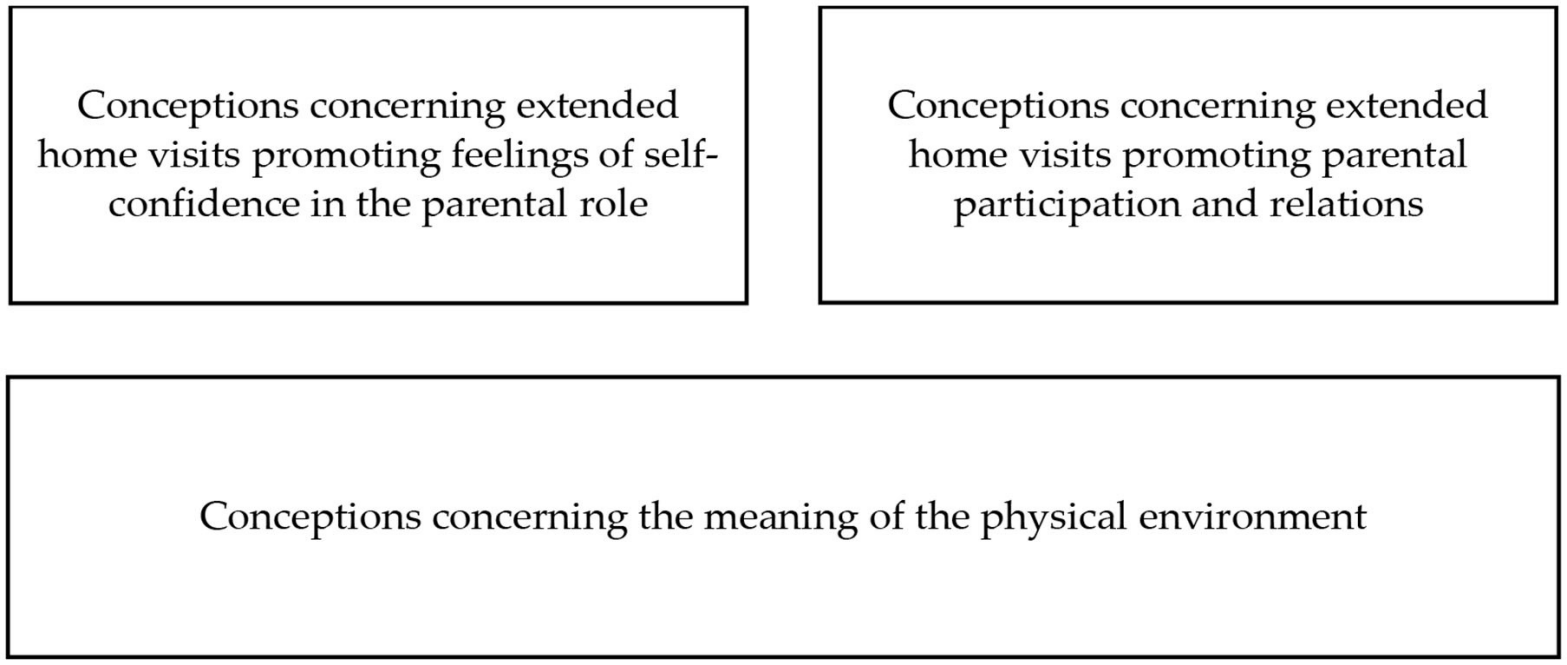
The findings in relation to the “outcome space,” and the hierarchical arrangement of the categories.

Using a phenomenographic approach allows for an analysis of the underlying structure of variance in the conceptions of and the relationship between the descriptive categories that arise during data analysis. This relationship is presented in an outcome space, where each descriptive category forms part of a larger whole. For this study, a hierarchical arrangement of the relationship between the descriptive categories arose and this arrangement illustrates the participants' understanding of their experiences of receiving professional support through extended home visits during pregnancy and the first 15 months of parenthood. The hierarchical arrangement—shown in Figure 1—is based on the theoretical assumption described below.

According to the participants' understanding of their experiences of receiving professional support through extended home visits during pregnancy and the first 15 months of parenthood, the descriptive category *conceptions concerning the meaning of the place of the meeting* formed the basis within the hierarchical arrangement. The participants considered the home to be a safe place in which to meet the professionals and this affected the entire family. The extended home visits facilitated the participants' feelings of manageability, meaningfulness, and a sense of coherence. From a theoretical perspective, such feelings promoted the participants' feelings of strengthened parenthood as well as their parental participation and relations. Subsequently, the second (*conceptions concerning extended home visits promoting feelings of strengthened parenthood*) and third (*conceptions concerning extended home visits promoting parental participation and relations*) descriptive categories were placed above the first descriptive category in the figure that illustrates the outcome space (Figure 1).

## Discussion

### Home as a Safe and Natural Meeting Place

The results from our study reveal that the place in which care meeting occur, as well as their form, seem to influence how parents perceive meaningfulness and manageability of their own parenthood, which also seems essential for their conceptualization of the support they were offered. Parents described that their home environment provided them with a sense of security, and the meaning of the meeting place was revealed to be the basis of the outcome space of our results. This is in line with earlier research that had described home visits as supporting parents in their parental role because support is applied in a natural environment (i.e., the parents' home) (4). Leirbakk et al. (7) pointed out that home visits increase the professionals' understanding of how families handle the challenge of parenthood. Other studies describe the importance of home visits to support the family in lifestyle matters because parents play an important role in children's health (9). Barboza et al. (4) also described home visits as a way to strengthen relationships within a new family. In line with this, Turnbull et al. (37) described the importance of securing the social situations of families so that they can receive support and be strengthened in their parental role. It is important to remember that, while the results of our study indicate that the home is a safe meeting place, this may not always be the case (e.g., in cases of domestic abuse). However, home visits may offer professionals a broader arena for identifying and supporting the parents and children in these situations, which could be a reason why home visits reduce the incidence of child abuse (11).

The parents participating in our study described that their home meant security for them. In addition, it also meant that the child had a natural place during in the care meeting, which was viewed as essential by parents. Their parental role, as well as the support they needed, became more apparent, positively influencing both parents and children. In line with this, Barboza et al. (4) described that home visits and the home environment are essential aspects of the support provided by professionals for new parents in their parental role. This is because professional support can then be provided in the natural environs of families. Studies have also described home visits as promoting the parents' ability to ask questions in contrast to meetings at healthcare centers (7, 10), increasing their support concerning their babies' needs (10). This is in line with Rautio (8), who described home visits as providing a basis for safe and trustworthy relationships with professionals. Parents felt heard and expressed that professionals had more time in their meetings, giving them a safe arena in which to ask questions. Leirbakk et al. (7) pointed out that all parents need professional support and that seemingly well-functioning parents also need an arena in which they can ask questions. These results point out the importance of creating a space in which parents feel safe to ask any question that will help them develop their parental role. This also implies that extended home visits represent important interventions that should be provided as a regular part of care during pregnancy and early childhood in order to promote self-confidence in parents because this is a challenging time in the life of a parent.

### Home Visits With a Focus on the Whole Family

Extended home visits involve an increased focus on the whole family. In the current study, the information that the parents received was described as being appropriately adapted and in line with the family's needs and situation. It was essential for the parents that they also received the information they needed in advance so that they could prepare. These results are in line with Barimani et al. (38), who stated that the relaxed attitude of professionals in meetings promotes the parents' ability to receive the support provided. Their results also stressed the importance of professionals having the time and ability to act reassuringly, allowing parents to raise uncomfortable issues. This, in turn, enabled professionals to provide support in accordance with the unique needs of parents. Reticena et al. (23) described a variety of professional support dimensions, such as promoting the development of the parental role, creating a safe environment for their children's upbringing, and providing guidance in life as parents. Together, these results stress the importance of support for both parents, which is in line with previous research that highlights the value of individual parent communication with the non-birthing parent to support family health and the child's upbringing (39). When planning future interventions for expectant and new parents, both the form of professional support and the types of the actual professionals involved in the provision of this support need to be taken into consideration. In the *Reinforced Parenting*—*Extended Home Visits* intervention, the extended home visits provided a safe place for parents and an arena in which the professionals were allowed to listen to parents in order to understand their life circumstances better.

### Home Visits Promoting Self-Confidence in Parenting

In our study, the participating parents noted that their self-confidence in relation to parenting was strengthened through the reassurance and safety experienced during the extended home visits. They described feeling well-informed and prepared in their parental role, secure in the idea that they could access professional support when they needed it. This is in line with other studies (4, 40) that reported that home visits lead to increased security in the parental role, increased trust in healthcare professionals, and a decrease in visits to the casualty department. In our study, parents also described extended home visits as a form of professional support that was individualized and reassuring. The home visits established a calm atmosphere that helped parents speak their thoughts. MacKenzie Bryers and van Teijlingen (41) and Wiklund et al. (42) described the importance of professional support adapted to the needs of parents and children and their unique situations. They stressed the importance of having professional support built on continuity. Such individualized support is essential for strengthening the parents' sense of security and safety during pregnancy and in early parenthood because this time of life is challenging. However, professional support should also aim to strengthen social support (43) and, in our study, home visits were also found to promote the idea of parents joining activities with other parents within CHC, thus promoting social support as well. Put together, it is essential to consider these results when care and support for new parents and their children are planned. Extended home visits and support in the parental home environments are likely to strengthen the parents on a more comprehensive level than the results of our study show. For the parents' satisfaction, it is essential that they feel a well-established sense of security in their current situation because becoming a parent is a challenging life event.

### Home Visits Creating Mutual Trust Between Parents and Professionals

The results of our study revealed that parents conceptualized the extended home visits as society's interest in and engagement with them as parents and as a family. The home visits were described as a crucial form of professional support, something all parents should be offered. For migrant parents, the extended home visits were seen as helping them with their integration into Swedish society. The parents also described the extended home visits as a form of replacement for the social support they lacked as a result of family and friends living far away. This is in line with Rautio (8), who described that parents need to have a trustworthy and safe relationship with professionals. Burström et al. (44) stated that support from and building relations with professionals are important measures for integration—which means that it is very important to offer extended home visits to migrant parents during pregnancy and early parenthood. In particular, our results show that extended home visits especially promoted social support for migrant parents.

The parents in our study described that a close and more equal relationship was created between them and the professionals through the extended home visits. They also described that increased participation and presence was created. Stubbs and Achat (6) pointed out the importance of the early identification of needs for extended support in order to promote and strengthen the relationship between parents and children, within the family as a whole, and between parents and professionals. Turnbull et al. (37) described home visits as a tool that can be used to gain a deeper understanding of a family's social situation and to secure it. This is in line with Marttila et al. (40), who related the experience of social workers, concluding that home visits allowed them to become better acquainted with new parents and their children, creating a sense of mutual trust. This could be important if contacts were needed later on in a child's life. In our study, parents did not differentiate between the different job functions of the professionals during home visits, which is essential because parents may hesitate to contact social workers but a visit from a midwife or CHC nurse is expected for childbirth and early parenthood in Sweden. This is especially important because social services provide support, such as family counseling, if parents are having difficulties in their relationships.

### Home Visits Promoting Parents' Meaningfulness and Manageability

The results of our study reveal that parents experienced meaningfulness and manageability of parenthood when professional support was provided in their homes. The participants pointed out that the professional support was more evident and that it felt more real when their parental role was actualized in their home environment, which meant that the parents could anchor the support to their lives and proper context. These results are in line with Bäckström et al. (18), who described how a sense of coherence can strengthen the quality of couple relations, which can affect the parents' ability to handle stressful situations and challenges in life. According to Mansfield et al. (45), this ability could be understood as resilience, which refers to a person's ability to handle stress, demands, and emotions as well as to solve problems and be adaptable. According to Masten and Barnes (17), resilience is a long-term ability that aids people in handling changes and continuing to develop. Mansfield et al. (45) conveyed that resilience refers to being able to seek and accept support and to having the ability to create and promote relations. Many of the experiences described by parents in our study could be understood as resilience factors that would strengthen and protect both the parents and the children in a family. The parents noted the importance of social support, of their care for their children, and of receiving support in their parental role during their children's upbringing. They also stressed the importance of professional support concerning parenthood, the child, and society. Earlier research has shown that positive parenthood can be understood as a resilience factor, together with engaged parents and social support in a variety of forms such as family, friends, and society (46). According to Seay et al. (13), positive parenthood facilitates the parents' ability to unconditionally meet the needs of their child. These researchers defined positive parenthood as a continuing relationship between the parents and children that includes care, nurture, teaching, training, leadership, and communication. Earlier research thus strengthens the importance of the results obtained in our study regarding the parents' experiences of extended home visits. Fritz et al. (46) showed that interventions that enhance the life situations of individuals and families—in combination with social support and resilience factors—can contribute to preventing difficulties during childhood. Aronen and Arajarvi (3) stressed the importance of early interventions for promoting health in children, with extended home visits being one example of such early interventions. The results of our study suggest that the parents experienced extended home visits as a form of professional support that strengthened their abilities to fulfill their parental role. It was essential for them to receive individualized support concerning their specific situations. Professional support for parents needs to adapt to the needs of today's parents.

A limitation of our study could be that some of the interviews were conducted in Arabic, which required an interpreter to be present. Performing interviews with an interpreter could affect the quality of the data. However, our data were rich and varied and provide results that increase our understanding of the parents' experiences of extended home visits. The number of participants was deemed to be satisfactory because our informants had diverse characteristics in terms of age, gender, education level, country of origin, and number of children, which is a strength of our study (47). The analysis focused on parents' understanding of their experiences of receiving professional support through home visits, and parents' socioeconomic status, gender, birth country, and parity were not generally considered, which might be a limitation in our study. Also, further exploration is needed regarding migrant families' needs and understanding of professional support. Phenomenography was considered to be a useful method for this study because it provided us with a broader understanding of the parents' experiences of extended home visits as a phenomenon (34). Another strength of our study was that the analysis was performed in a team and that all authors were engaged in its final steps. However, further studies that utilize various methods are needed in order to further deepen our understanding and develop knowledge of the impacts and benefits of home visits for both parents and children.

### Conclusions and Clinical Implications

Extended home visits—as a form of professional support—appear to promote the self-confidence of parents in their parenting ability, giving them a feeling of security that facilitates conversations with professionals. Moreover, parents feel that their children behave more characteristically during home visits because both the children and the entire family have a natural role in the home setting. The results of this study indicate that professional support during home visits is conceptualized as reassuring and individualized, making it easier to understand information and receive support from professionals, thus improving social support through social activities at child health centers. Furthermore, it is also found that home visits may facilitate parental participation and integration into Swedish society for migrant parents. Cooperation between professionals is found to be important. Professional support should be adjusted to the unique individual needs of parents, which demands a variety of supportive interventions—for example, reorganizing one or two of the regular clinical visits currently being scheduled as home visits instead.

## Data Availability Statement

The datasets generated for this article are not readily available because we do not have permission to share data. Requests to access the datasets should be directed to caroline.backstrom@his.se.

## Ethics Statement

The studies involving human participants were reviewed and approved by the Regional Ethical Review Board in Gothenburg (Dnr 2019:03906). The patients/participants provided their written informed consent to participate in this study.

## Author Contributions

CB contributed to the study's conceptualization, formal analysis, investigation, methodology, validation, and visualization, as well as to writing the article, which is an original draft. A-CF and JP contributed to the conceptualization, data curation, formal analysis, funding acquisition, investigation, methodology, project administration, resources, software, validation, visualization, and writing of the article. ST and ML contributed to the conceptualization, formal analysis, validation, and writing of the article. All authors contributed to the article and approved the submitted version.

## Conflict of Interest

The authors declare that the research was conducted in the absence of any commercial or financial relationships that could be construed as a potential conflict of interest.
